# NF2 and Canonical Hippo-YAP Pathway Define Distinct Tumor Subsets Characterized by Different Immune Deficiency and Treatment Implications in Human Pleural Mesothelioma

**DOI:** 10.3390/cancers13071561

**Published:** 2021-03-29

**Authors:** Haitang Yang, Sean R. R. Hall, Beibei Sun, Liang Zhao, Yanyun Gao, Ralph A. Schmid, Swee T. Tan, Ren-Wang Peng, Feng Yao

**Affiliations:** 1 Department of Thoracic Surgery, Shanghai Chest Hospital, Shanghai Jiao Tong University, Shanghai 200030, China; haitang.yang@dbmr.unibe.ch; 2 Gillies McIndoe Research Institute, 6242 Wellington, New Zealand; sean.hall@gmri.org.nz (S.R.R.H.); swee.tan@gmri.org.nz (S.T.T.); 3 Institute for Thoracic Oncology, Shanghai Chest Hospital, Shanghai Jiao Tong University, Shanghai 200030, China; bbsun167@163.com; 4 Division of General Thoracic Surgery, Department of BioMedical Research (DBMR), Inselspital, Bern University Hospital, University of Bern, 3008 Bern, Switzerland; liang.zhao@dbmr.unibe.ch (L.Z.); yanyun.gao@dbmr.unibe.ch (Y.G.); Ralph.Schmid@insel.ch (R.A.S.)

**Keywords:** mesothelioma, NF2, LATS2, YAP, Hippo pathway, targeted therapy, immunotherapy

## Abstract

**Simple Summary:**

It is a long-held notion that loss-of-function mutations in negative regulators of the Hippo-YAP pathway, such as NF2, LATS1/2, have a similar potential to promote nuclear YAP activity, which is thought to play an essential role in the pathogenesis of MPM. Whether loss-of-function in these individual regulators uniformly affects the Hippo-YAP activity and contributes to a similar disease phenotype has not yet been revealed in MPM. Surprisingly and interestingly, we found in this study that loss-of-function in the upstream regulator NF2 of the Hippo pathway is linked to the aberrant activation of Hippo-YAP-independent signaling. More importantly, our work showed NF2 loss-of-function and dysregulated Hippo-YAP pathway define distinct MPM subsets that differ in molecular features, therapeutic implications, patients’ prognosis, and in particular, infiltrative immune signatures. Our findings in this study may be instrumental for the precise management of immunotherapy and/or targeted therapy for MPM patients.

**Abstract:**

(1) Inactivation of the tumor suppressor NF2 is believed to play a major role in the pathogenesis of malignant pleural mesothelioma (MPM) by deregulating the Hippo-YAP signaling pathway. However, NF2 has functions beyond regulation of the Hippo pathway, raising the possibility that NF2 contributes to MPM via Hippo-independent mechanisms. (2) We performed weighted gene co-expression analysis (WGCNA) in transcriptomic and proteomic datasets obtained from The Cancer Gene Atlas (TCGA) MPM cohort to identify clusters of co-expressed genes highly correlated with NF2 and phospho (p)-YAP protein, surrogate markers of active Hippo signaling and YAP inactivation. The potential targets are experimentally validated using a cell viability assay. (3) MPM tumors with NF2 loss-of-function are not associated with changes in p-YAP level nor YAP/TAZ activity score, but are characterized by a deficient B-cell receptor (BCR) signaling pathway. Conversely, MPM tumors with YAP activation display exhausted CD8 T-cell-mediated immunity together with significantly upregulated PD-L1, which is validated in an independent MPM cohort, suggesting a potential benefit of immune-checkpoint inhibitors (ICI) in this patient subset. In support of this, mutations in core Hippo signaling components including LATS2, but not NF2, are independently associated with better overall survival in response to ICI in patients. Additionally, based on cancer cell line models, we show that MPM cells with a high Hippo-YAP activity are particularly sensitive to inhibitors of BCR-ABL/SRC, stratifying a unique MPM patient subset that may benefit from BCR-ABL/SRC therapies. Furthermore, we observe that NF2 physically interacts with a considerable number of proteins that are not involved in the canonical Hippo-YAP pathway, providing a possible explanation for its Hippo-independent role in MPM. Finally, survival analyses show that YAP/TAZ scores together with p-YAP protein level, but not NF2, predict the prognosis of MPM patients. (4) NF2 loss-of-function and dysregulated Hippo-YAP pathway define distinct MPM subsets that differ in their molecular features and prognosis, which has important clinical implications for precision oncology in MPM patients.

## 1. Introduction

Malignant pleural mesothelioma (MPM) is a highly lethal cancer, predominantly characterized by the inactivation of tumor suppressor genes (TSGs) [1,2]. Large-scale cancer genome sequencing of human MPM samples reveals that TSG genes involved in the Hippo signaling pathway are mutated at a high frequency [3,4,5].

The core components of the Hippo signaling pathway include MST1 (mammalian STE20-like protein kinase 1) and MST2, large tumor suppressor kinase 1/2 (LATS1/2), and two adaptor proteins SAV1 (Salvador homolog 1), MOB1A/B (MOB kinase activator 1A/B) [6]. Hippo signaling converges on the LATS1/2-dependent phosphorylation of the transcriptional master YAP (Yes-associated protein, encoded by YAP1). LATS kinase-mediated phosphorylation at multiple sites (e.g., Ser127, Ser109) leads to sequestration within the cytoplasm due to 14-3-3 protein binding followed by ubiquitin-mediated proteolysis, negatively regulating YAP transcriptional activity. Following inactivation of the Hippo pathway, unphosphorylated YAP and its co-activator TAZ translocate to the nucleus and bind to TEAD1–4 transcription factors to control the expression of their target genes, which have been shown to control cell proliferation and inhibit cell death, underpinning the tumorigenic potential of YAP/TAZ. The core kinase Hippo pathway components are regulated by numerous upstream proteins, e.g., neurofibromin 2 (NF2), TAOKs and KIBRA, thereby linking the Hippo pathway to multiple aspects of cancer, such as cell size and proliferation, tissue regeneration, immunity, metabolism, epithelial-to-mesenchymal transition (EMT), and cancer therapy resistance and metastasis [7]. Of note, loss-of-function mutations in NF2 and LATS1/2 have been frequently observed, accounting for approximately 50% of MPM cases [3,4]. In addition, inactivation of the Hippo pathway can also result from non-mutational events, such as post-translational modifications [8].

We have recently systematically analyzed the potential therapeutic targets associated with genetic alterations affecting the major TSGs in MPM [3], which suggests that two well-characterized upstream components of the Hippo pathway NF2 and LATS1/2 that negatively regulate YAP activity [9], may have different roles in MPM. Despite the high prevalence of the dysregulated Hippo pathway in MPM, the role of these individual components in disease pathogenesis is not completely understood. In addition, it remains unclear whether NF2, the upstream regulator, mainly functions through the canonical Hippo-YAP pathway or confers additional molecular rewiring during the development of MPM.

In this study, we implemented weighted gene co-expression network analysis (WGCNA) [10], a robust algorithm to uncover molecular rewiring correlated with phenotypic/genotypic traits of interest, to identify biologically meaningful clusters (modules) of interconnected genes highly correlated with NF2 and p-YAP protein level, a surrogate marker of YAP inactivation and active Hippo signaling, in MPM patients. WGCNA was performed in proteomic datasets that are usually of higher reliability than transcriptomics in reflecting signaling activity mediated by the pathway of interest obtained from The Cancer Gene Atlas (TCGA) MPM cohort. We found different molecular signatures, particularly involving the tumor immune microenvironment and therapeutic vulnerabilities specifically associated with NF2 deficiency and canonical Hippo-YAP pathway dysregulation. Our results revealed previously underappreciated roles of NF2 independent of canonical Hippo-YAP signaling pathway, different immune infiltrates specific to NF2 and Hippo-YAP signaling, and more importantly, supported further stratification of patients with MPM based on dysregulation of NF2 and Hippo-YAP.

## 2. Results

### 2.1. Molecular Clusters Correlated with NF2 and p-YAP Protein Levels in Clinical MPM Samples

**Figure 1:**
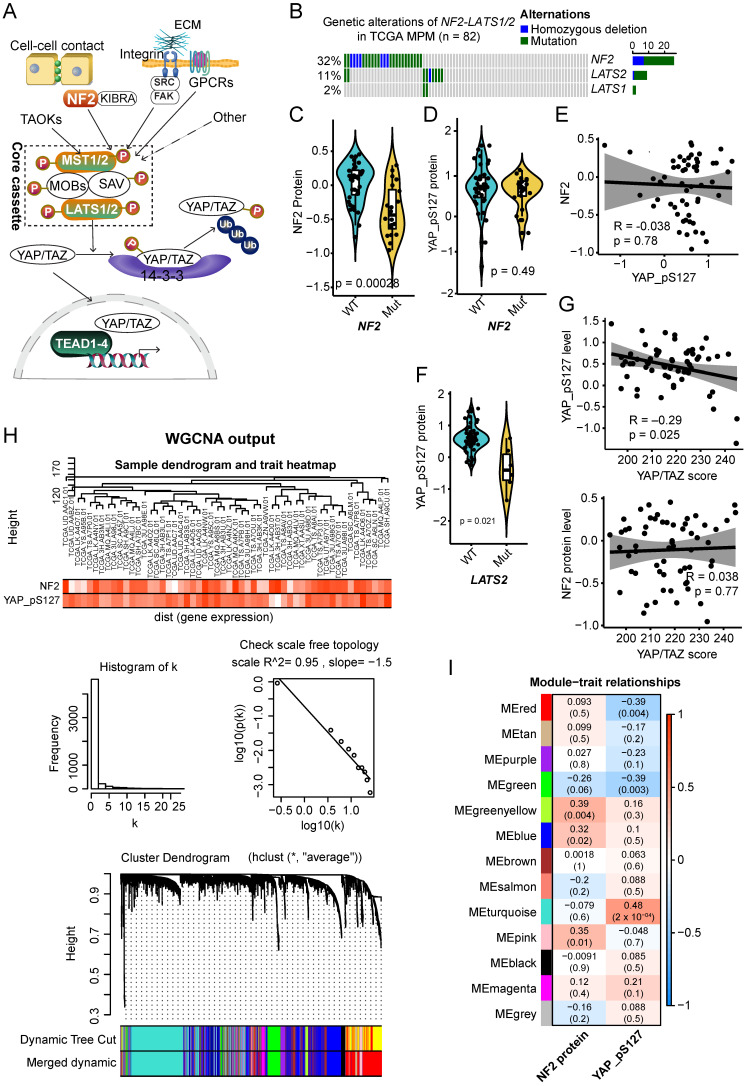
Weighted gene correlation network analysis (WGCNA) reveals gene modules or clusters correlated with NF2 and p-YAP in MPM. (**A**) Schematic representation showing key signaling molecules regulating Hippo pathway and YAP activity. NF2 and other proteins function upstream and facilitate the activation of MST1/2, and then activate LATS1/2, which causes inhibitory phosphorylation of YAP. Hypo-phosphorylated YAP translocates to the nucleus and recruits TEAD transcription factors that regulate various biological processes. MST1/2-LATS1/2 complex acts as a core component of the Hippo signaling pathway. (**B**) Genetic alterations of Hippo pathway components (NF2, LATS1/2) in TCGA (The Cancer Genome Atlas) malignant pleural mesothelioma (MPM). (**C**,**D**) The protein level of NF2 and phospho (p)-YAP (S217) in MPM tumors with mutated (mut) and wild-type (WT) NF2. (**E**) The correlation between NF2 and p-YAP (S217) at the protein level in TCGA MPM. (**F**) The protein level of p-YAP (S217) in MPM tumors with mutated (mut) and wild-type (WT) LATS2. Note that the protein quantification of LATS2 is not provided in the Reverse Phase Protein Array (RPPA) dataset. (**G**) The correlation between YAP/TAZ score and p-YAP (S217) at the protein level in TCGA MPM. (**H**,**I**) WGCNA analysis. In (**H**) the upper panel shows the sample dendrogram and trait heatmap. The middle panel shows a histogram of network connectivity and the right is a log–log plot of the same histogram. The lower panel shows the gene dendrogram obtained by average linkage with hierarchical clustering. The color row underneath the dendrogram shows the module assignment determined by the Dynamic Tree Cut. Gray genes are unassigned to a module. Gene expression similarity is determined using a pair-wise weighted correlation metric, and clustered according to a topological overlap metric into modules. (**I**) Consensus network modules correlated with NF2 and p-YAP protein levels in MPM using the eigenmodule (the first principal component of the module). Pearson correlation coefficient along with *p*-value in parenthesis underneath; color-coded according to correlation coefficient (legend at right). The blue color indicates a negative correlation, while the red represents a positive correlation. WGCNA analysis of gene expression generated by unsupervised hierarchical clustering on the basis of topographical overlap followed by branch cutting reveals 13 network modules coded by different colors. Genes in the positively correlated modules (in red) indicate the abundance of these genes conferred by individual genetic events, while those in the negatively correlated ones (in blue) indicate the attenuation. Genes in the gray module are those that cannot be clustered.

Studies have suggested that NF2 suppresses tumorigenesis by activating core components of the Hippo pathway (Figure 1A) [11,12,13]. NF2 is frequently mutated in clinical MPM specimens (Figure 1B) and NF2 loss-of-function plays a key role in the pathogenesis of MPM [11,14]. To further explore this, we integrated the Reverse Phase Protein Array (RPPA) data (containing 220 proteins) from the TCGA cohort of MPM patients (*N* = 61; https://tcpaportal.org/tcpa/ accessed on 21 December 2020), which, as expected, showed that genetic alterations of NF2 results in a significant decrease in NF2 protein levels (Figure 1C). Despite this, the NF2 mutation is not associated with altered expression of phospho (p)-YAP (S127) (Figure 1D), a well-established surrogate marker indicative of the activity of the Hippo signaling pathway. Along these same lines, we did not observe a correlation between NF2 and p-YAP (S127) at the protein level (Figure 1E). These results suggest that the genetic alteration of NF2 does not significantly affect the canonical Hippo-YAP pathway in MPM. In contrast, genetic alterations of LATS2, a core component of the Hippo pathway, caused a significant downregulation of p-YAP (S127), indicating compromised Hippo signaling induced by LATS2 loss-of-function (Figure 1F). Further, we utilized a previously curated YAP-TAZ gene signature that integrated target genes specific to the YAP-TAZ pathway [15], which demonstrated that p-YAP (S127), but not NF2, was significantly correlated with the YAP-TAZ score (Figure 1G). Taken together, our analysis showed that, unlike LATS2, NF2 might play additional roles, independent of the canonical Hippo-YAP pathway in the complex pathogenesis of MPM.

Of note, previous evidence demonstrated that Hippo-YAP activity could be regulated by other signaling pathways beyond the LATS1/2 core cassette [16], and non-mutational mechanisms could contribute to a deficient NF2 and core Hippo-YAP pathway [8,17]. We also observed high heterogeneity of NF2 protein levels in NF2-wild-type samples (Figure 1C), and of DNA methylation in NF2, LATS1/2, and YAP1 across MPM tumors (Figure S1A). As such, proteomic data (e.g., NF2, p-YAP) may more reliably reflect signaling pathway (e.g., NF2, canonical Hippo) activity compared with transcriptomics and genetic alterations. Thus, we utilized protein levels of NF2 and p-YAP to reflect the functional activity of the corresponding pathways.

To uncover molecular features associated with NF2 and canonical Hippo-YAP pathway in MPM, we applied weighted gene co-expression network analysis (WGCNA) [9,10], an algorithm to cluster molecular aberrations that correlate with NF2 protein and p-YAP (S127), to the RPPA and whole-genome transcriptomic data of the TCGA MPM cohort (Figure 1H,I). The correlation analysis revealed 13 molecular modules or clusters of genes color-coded according to the convention of WGCNA that are positively or negatively correlated with NF2 or p-YAP (S127) (Figure 1I). Notably, genes in the positively correlated modules indicate their abundance (co-expression) with respect to NF2- and p-YAP-represented samples, while the negatively correlated ones signify attenuation of the network of genes. Genes in the gray module are unable to be clustered [9]. The dysregulated molecular modules specific to NF2 and canonical Hippo-YAP revealed by robust WGCNA would provide valuable information on their individual roles in the pathogenesis of MPM.

### 2.2. NF2 Loss-of-Function Is Characterized by a Deficient B-Cell Receptor (BCR) Signaling Pathway

The correlation network identified three significant modules/clusters that were all positively correlated with NF2 protein levels (Figure 1I), meaning that the expression of the genes in the three modules is low in MPM samples with an NF2 defect. The most correlated module is the greenyellow module, containing 482 genes (correlation coefficient Pearson’s r = 0.39; *p*-value = 0.004), followed by the pink (351 genes; r = 0.35; *p*-value = 0.001), and the blue (742 genes; r = 0.32; *p*-value = 0.02). Pathway analyses (GO, KEGG, Reactome) revealed that the genes enriched in the greenyellow module are mainly involved in several pathways, e.g., MAPK signaling, proinflammatory signaling (Figure S2A).

**Figure 2:**
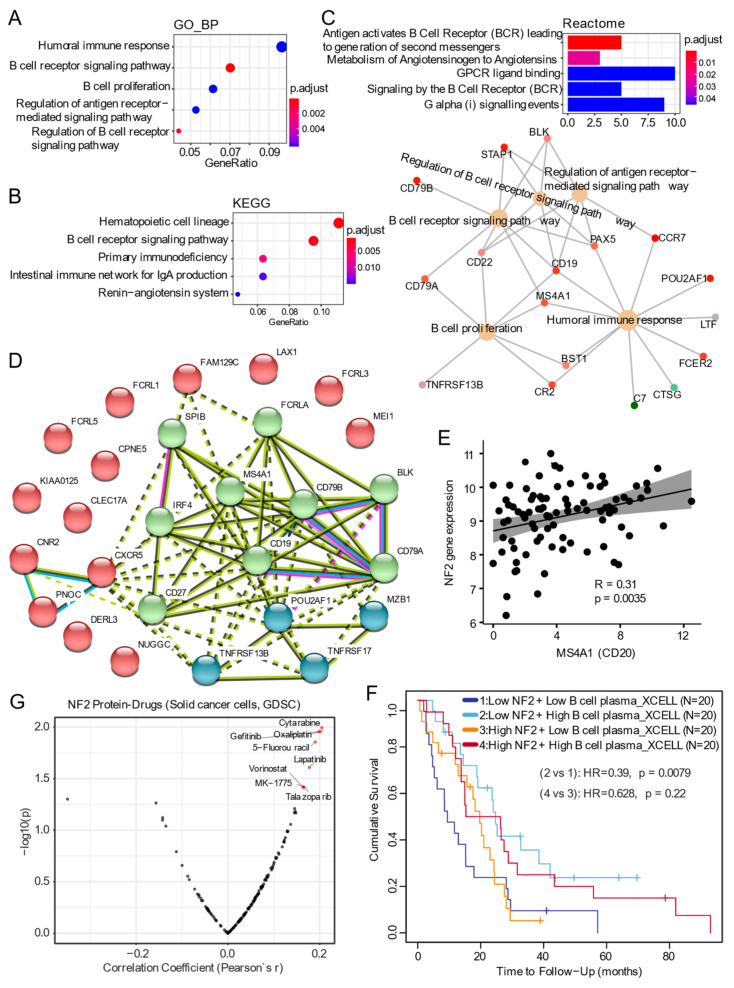
NF2 loss-of-function is characterized by a deficient B-cell receptor (BCR) signaling pathway. (**A**–**C**) The top 5 significantly enriched Gene Ontology (GO; biological process (BP)), Kyoto Encyclopedia of Genes and Genomes (KEGG) (**B**), and Reactome (**C**) pathways based on genes in the MEpink module. Cnetplot in (**C**, lower panel) list genes in the enriched Reactome pathways. (**D**) STRING (https://string-db.org/ accessed on 21 December 2020) protein interaction map based on the top 30 hub genes in the MEpink module. (**E**) Correlation (Pearson r) between gene expression of CD20 (also known as MS4A1), a B-lymphocyte-specific membrane protein, and NF2 across TCGA MPM cohort. (**F**) Kaplan–Meier curves for MPM patients grouped by the plasma B-cell infiltrative signature and NF2 gene expression. The high vs. low groups are based on the median value of the indicated gene expression. Here, multiple covariates, e.g., tumor stage and purity, patients’ age and gender were included for adjustment. (**G**) Volcano plot showing the correlation between the AUC (Area the Under Curve) value of drugs and the NF2 protein level. Blue dots indicate the significantly (*p*-value < 0.05) negatively correlated drugs while the red indicates the positively correlated ones. Here, a positive correlation indicates the association of a larger (more resistant) AUC value with higher gene expression and vice versa.

Intriguingly, the pink module was significantly enriched for genes of the BCR signaling pathway, indicating a potential loss of antibody-mediated humoral immunity in NF2-deficient MPM (Figure 2A–C). In addition, we analyzed the intramodular connectivity, given that highly connected genes within a single module may serve as the hub with core regulatory roles. The top 30 most connected genes in the pink module (ranked by connection degree (from high to low)) were: *CD79A, CD19, FCRL5, MGC29506, CD27, KIAA0125, CD79B, ADAM6, TNFRSF17, MS4A1, FCRLA, PNOC, LOC96610, POU2AF1, LAX1, C8orf80, BLK, CPNE5, CXCR5, CNR2, TNFRSF13B, FCRL1, IRF4, FLJ40330, CLEC17A, FCRL3, MEI1, DERL3, FAM129C,* and *SPIB* (Figure 2D). Among these hub genes, POU2AF1 is a transcriptional coactivator that is essential for the response of B-cells to antigens and is required for the formation of germinal centers. The transcription factor SPIB binds to the PU-box, a purine-rich DNA sequence acting as a lymphoid-specific enhancer [18,19]. Genes in the blue module were enriched in a stromal signature, e.g., angiogenesis, extracellular matrix organization, extracellular ligand-receptor interaction, and mesenchymal signature (Figure S2B), suggesting that MPM patients with NF2 loss-of-function might not be responsive to anti-angiogenic therapies, which have shown a highly heterogeneous effect in unselected MPM patients [20,21].

Given that our above WGCNA analysis revealed a potential loss of antibody-mediated humoral immunity in NF2-defective MPM (Figure 2A–C). We further analyzed the correlation between gene expression of CD20 (also known as MS4A1), a B-lymphocyte-specific membrane protein [22,23], and NF2 across TCGA MPM samples, demonstrating a significantly positive correlation pattern (Figure 2E). Recent evidence highlighted the previously underappreciated role of intra-tumoral B-cells in predicting patients’ prognosis and mediating therapeutic responses to immune checkpoint blockade (ICB) treatment [24,25]. Interestingly, we observed that in MPM with low NF2 expression, a high plasma B-cell infiltrative signature predicts better overall survival (Figure 2F).

Concerning the potential therapeutic targets associated with NF2 loss-of-function in tumors, we correlated the protein level of NF2 with the drug sensitivity profiles across a panel of solid cancer cells based on the publicly available GDSC dataset (https://www.cancerrxgene.org/, accessed on 21 December 2020). Our analysis revealed that cancer cells with decreased NF2 expression display high sensitivity to DNA synthesis inhibition (Cytarabine, Oxaliplatin, and 5-Fluorouracil) [26] and epidermal growth factor receptor (EGFR) targeted therapies (Gefitinib and Lapatinib) [27,28,29] (Figure 2G). In support of this, previous evidence showed that NF2 negatively regulated EGFR signaling by restraining the EGFR into a membrane compartment, preventing the signaling transduction or internalization of EGFR. In confluent Nf2 −/− cells, EGFR activation persists, driving continued proliferation that is halted by specific EGFR inhibitors [27]. Whether the sensitivity to EGFR-targeted therapies is observed in immune-competent in vivo models or clinical settings awaits further investigation.

### 2.3. MPM Tumors with Lower p-YAP Level Display an Exhausted T-Cell-Mediated Immune Phenotype

In view of the role of the canonical Hippo pathway in MPM, we observed that the inactive form of YAP, p-YAP (S127), was positively correlated with the turquoise module (1084 genes; r = 0.48; *p*-value = 2 × 10^−4^), but negatively with the green (482 genes; r = −0.39; *p*-value = 0.003), followed by the red (476 genes; r = −0.39; *p*-value = 0.004) (Figure 1I). Genes in the turquoise module are mainly enriched for the neuronal system, keratinization, the formation of the cornified envelope, and EGFR signaling pathways (Figure S3A), suggesting a reduced expression of these genes in MPM samples with YAP activation. Of note, the latter three signaling pathways are mainly related to the epithelial phenotype, which was in agreement with previous reports demonstrating that YAP is a critical mediator of the EMT process and mediates the resistance to EGFR-targeted therapies [30,31].

Genes in the negatively correlated green module are predominantly involved in cell division, particularly mitosis (Figure S3B), suggesting a high tumor proliferation activity in MPM with YAP activation that is consistent with the canonical roles of the Hippo-YAP pathway in regulating cell size and proliferation [32]. Thus, inhibitors (e.g., AURKB/AURKA inhibitor; Figure S3B) targeting the mitotic phase may be promising for this MPM subset, as direct targeting of YAP is highly toxic and there are no clinically approved specific inhibitor directly blocking YAP signaling [33]. Previous evidence also reveals the importance of targeting AURKB/AURKA in cancer cells with activated YAP [34,35].

**Figure 3:**
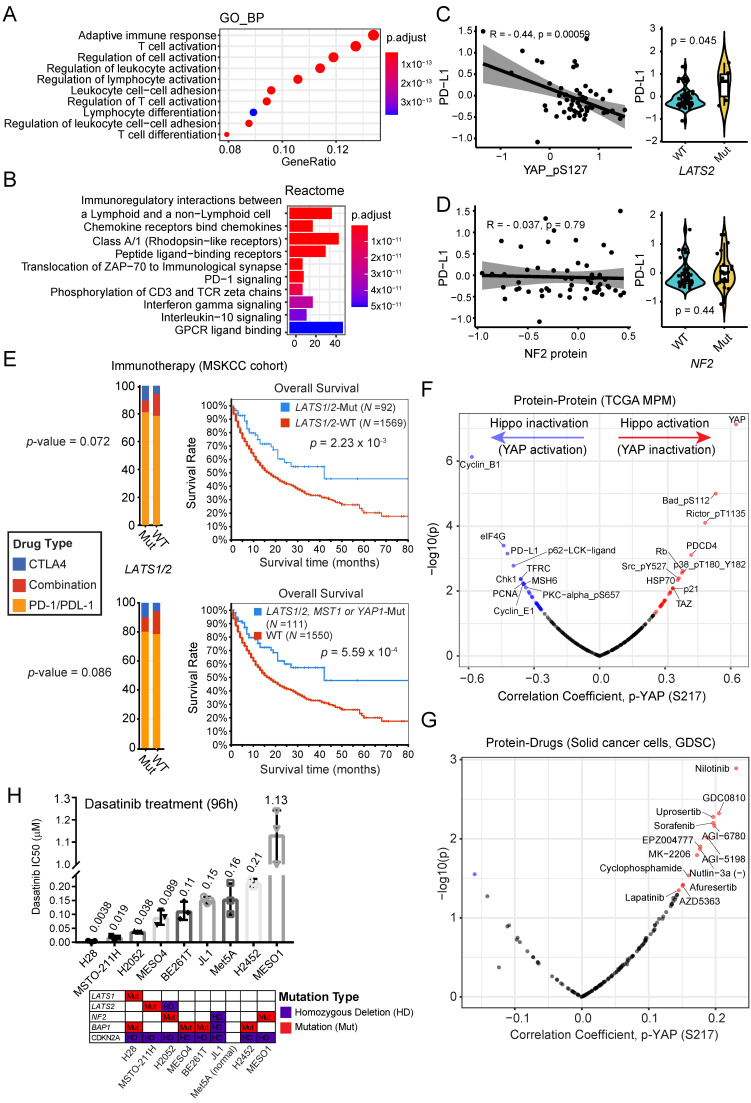
MPM tumors with YAP activation display exhaustion of T-cell-mediated immune response. (**A**,**B**) The top 10 significantly enriched Gene Ontology (GO; biological process [BP]) (**A**) and Reactome (**B**) pathways based on genes in the MEred module. (**C**,**D**) Correlation (Pearson r) between PD-L1 protein and p-YAP (**C**, left) or between PD-L1 protein and NF2 protein (**D**, left). Violin plots show the association between PD-L1 and the genetic alterations of LATS2 (**C**, right) or NF2 (**D**, right). (**E**) Association between the mutations in LATS1/2 (*n* = 92) or other key regulatory components (LATS1/2, MST1, YAP1; *n* = 111) of the Hippo pathway with overall survival in cancer patients after immune checkpoint blockade (anti-PD-1/PD-L1, or anti-CTLA4, or combination treatment). Of note, there is no significant difference in the treatment regimens between the wildtype (WT) and mutated (Mut) subgroups. Data were downloaded from cBioPortal https://www.cbioportal.org/ accessed on 21 December 2020. (**F**,**G**) Volcano plots showing the proteins (**F**) and drugs (**G**) that are correlated with p-YAP across TCGA MPM samples and solid cancer cell lines, respectively. Blue dots indicate the significantly (*p*-value < 0.05) negatively correlated proteins/drugs while the red the positively correlated ones. In (**G**) the AUC (Area Under Curve), value of drugs was used to indicate the drug effect, with a positive correlation representing the association of a larger AUC value (more resistance) with a higher p-YAP level and vice versa. (**H**) The median inhibitory concentration (IC50) values of a panel of MPM cell lines were treated with Dasatinib (96 h). MPM cells seeded in triplicate at 96-well plates were drugged 24 h later, over a 12-point concentration range (two-fold dilution). DMSO-treated cells were used as control. IC50 was determined using GraphPad Prism 7. *N* = 3 biological replicates. The lower panel shows the gene annotations of the indicated MPM cell lines.

Of particular interest, genes in the negatively correlated red module were mostly enriched in T-cell-mediated adaptive immune responses (Figure 3A,B). The top 30 most connected genes in the red module (ranked by connection degree (from high to low)) were: *LCK, CD3E, SH2D1A, CD6, CD3D, SIRPG, CD2, SLAMF6, IL2RG, CD247, TBC1D10C, TRAT1, SIT1, ZAP70, CXCR3, SLA2, ITGAL, CD5, GZMK, TIGIT, CD96, ZNF831, ITK, THEMIS, PYHIN1, P2RY10, ACAP1, CXCR6, CD27, GPR171*. Among these, TIGIT represents an immune checkpoint T-cell immunoreceptor overexpressed by T-cells with an exhausted phenotype and functions via suppressing T-cell activation by promoting the generation of mature immunoregulatory dendritic cells, and its inhibitors have shown promises in clinical trials [36]. Additionally, we observed a significant negative correlation between PD-L1 and p-YAP at the protein level in MPM, and *LATS1/2*-mutant MPM tumors were associated with higher PD-L1 expression (Figure 3C). These data indicate that a mix of activated and exhausted T-cells in MPM samples are associated with activated YAP signaling. In contrast, there was no correlation between PD-L1 and NF2 at the protein level and also no significant difference in the PD-L1 protein level between MPM tumors with mutated and wild-type NF2 (Figure 3D). These results further support the differential roles of NF2 and Hippo pathways in the pathogenesis of MPM. Indeed, a retrospective analysis of patients receiving ICB [37] demonstrated that mutations of LATS1/2, rather than of NF2, predict better overall survival, which was even more evident after incorporating MST1 and YAP1 mutations (Figure 3E). Collectively, these data highlight the importance of ICB treatment for a subset of MPM patients with an inactivated Hippo pathway.

Besides, we sought to know whether the correlation between YAP activity and PD-L1 expression is specific to MPM tumors. Based on the TCGA Pan-cancer cohort, we observed that the correlation pattern between YAP activity (reflected by curated YAP/TAZ downstream target score) and PD-L1 expression displays the highest in MPM (Figure S4A), suggesting that tumor-derived microenvironment may play a role in regulating PD-L1 in addition to YAP signaling. Supporting this notion, MPM tumors display the highest YAP/TAZ downstream target score across different cancer types (Figure S4B).

In addition, at the protein level, we identified Cyclin B1, a key regulator of the cell cycle at the G2/M (mitosis) transition, as the most negatively correlated protein with p-YAP (S217), which is in agreement with the aforementioned green module that is predominantly involved in the mitotic process (Figure 3F). Moreover, we integrated the drug sensitivity profile (indicated by Area Under the Curve (AUC), higher AUC suggests more resistance) and proteomic data across hundreds of solid cancer cell lines, demonstrating that cells with high YAP activity (reflected by low p-YAP) are most sensitive to Nilotinib, a clinically approved BCR-ABL/SRC inhibitor (Figure 3G). To test this, we treated a panel of MPM cells with Dasatinib, another clinically approved inhibitor selectively targeting BCR-ABL and SRC, which showed that MPM cells with *LATS1/2*-mutation are most sensitive to Dasatinib (Figure 3H), as MPM tumors with *LATS1/2* mutations were associated with significantly higher YAP (Figure 1F). Immunoblot data showed that *LATS1/2* mutations did not result in loss of protein expression (Figure S5), which was in line with a previous study by Miyanaga et al. [38] This observation might be because mutated LATS1/2 affects its kinase function but not the protein level, or because there exists some other molecular aberrations that affect the Hippo-YAP signaling pathway in these cell lines. For instance, MESO-211H cell line harbors *LATS2* mutation but not *LATS1*; however, evidence shows the presence of fusion transcripts of *LATS1* in MESO-211H, which leads to its functional loss [38]. Interestingly, preclinical and clinical evidence demonstrated that BCR-ABL/SRC inhibitors enhance the cytotoxic effects of T-cells and the efficacy of ICBs [9,39,40], suggesting that BCR-ABL/SRC inhibitors not only target the cancer cells but also modulate the tumor immune microenvironment. Notably, Met5A, which has been widely used as a normal mesothelial cell line, also exhibits a certain responsiveness to Dasatinib. This might result from the effect of the integrated SV40 Tag in Met5A cells [41], in that SV40Tag is one of the most potent carcinogens, and is known to cause massive aneuploidy due to its perturbation of the retinoblastoma (pRb) and p53 tumor suppressor proteins [42].

Notably, four highly selective AKT inhibitors (Uprosertib, AZD5363, Afuresertib, MK-2206) were also listed as potential sensitive inhibitors (Figure 3G). This observation was in line with the protein correlation data showing that increased YAP activity (lower p-YAP) is positively correlated with enhanced AKT signaling pathway, which is reflected by a high level of eIF4G (positive correlation), a key downstream effector of AKT/mTOR, and a low level of p-RICTOR (negative correlation), a well-established negative upstream regulator of AKT/mTOR (Figure 3F). In support of this, YAP has been shown to mediate the crosstalk between the Hippo and PI3K–AKT–mTOR pathway in organogenesis [43]. These data suggest that cancer cells with activated Hippo-YAP signaling may be more sensitive to AKT-targeted therapy. Given the potential roles of YAP in immunity, AKT inhibition would likely improve the sensitivity of tumors to the host immune system resulting from activated YAP signaling. Supporting this notion, targeting the PI3K-AKT-mTOR pathway has been shown to modulate the immunosuppressive microenvironment, enhancing the sensitivity to tumor-specific CD8+ T-cell-mediated cytotoxicity, and augmenting the effect of immunotherapy [44,45].

### 2.4. Dysregulation of NF2 and Hippo-YAP Exhibits Different Infiltrative Immune Signatures in MPM

**Figure 4:**
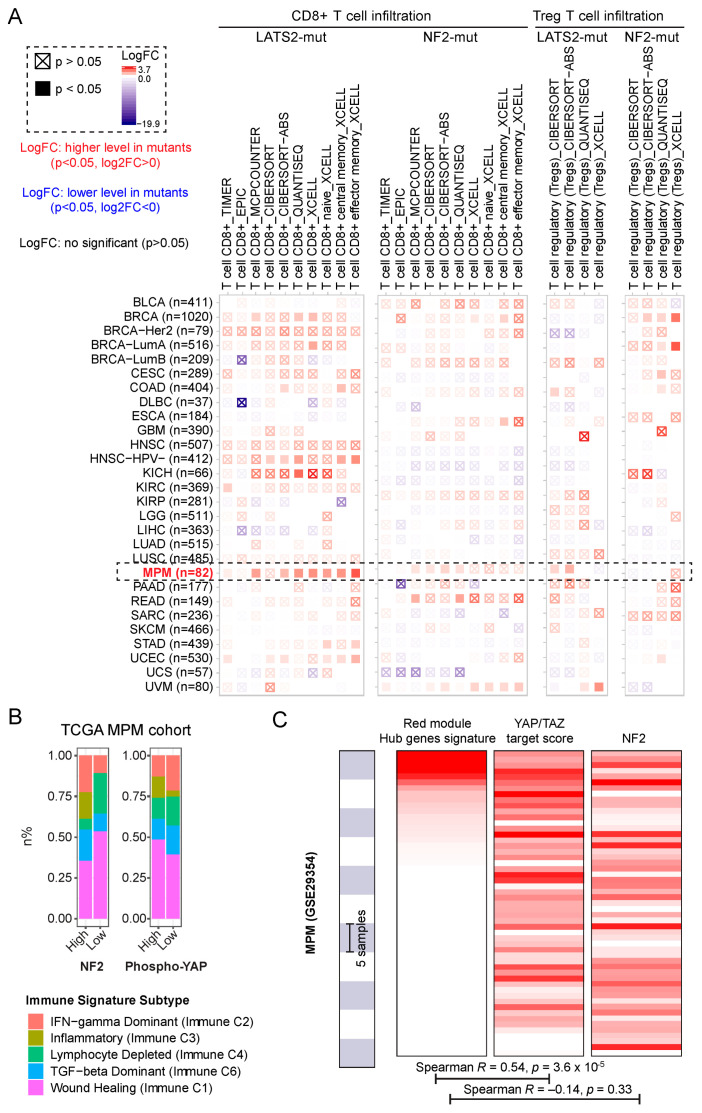
Dysregulation of NF2 and Hippo-YAP exhibits different infiltrative immune signatures in MPM. (**A**) Tumor-infiltrating immune cell profiles across the TCGA pan-cancer cohort were shown. The number of patients shown in parentheses. Data were downloaded from TIMER (version 2.0), a comprehensive resource for systematic analysis of immune infiltrates across diverse cancer types (http://timer.comp-genomics.org/) (See the methods). (**B**) Percentage of immune subtype models (C1–C6) across the TCGA MPM cohort, in which the reverse-phase protein array (RPPA) data were used. The genes contained in each signature were evaluated using model-based clustering by p the “mclust” R package. Each sample was finally to be grouped based on its predominance with the C1–C6 signature. The immune subtype models were based on Thorsson V et al. Immunity. 2018 (See the methods). (**C**) Correlative analysis of the hub genes-based signature score in the red module (Figure 1I) with YAP/TAZ target score and NF2 gene expression based on an independent MPM dataset (GSE29354).

Given the dysregulated immune profiles described above, we performed a systematic analysis of immune infiltrates across TCGA pan-cancer cohort (*n* = 28) in the context of mutated *NF2* and *LATS1/2*, based on the TIMER algorithm that provides information regarding immune cell types and their abundance by multiple immune deconvolution methods [46]. Our analysis revealed tumor-infiltrating CD8+ T-cells, particularly CD8+ effector memory T-cells, were mainly enriched in MPM harboring *LATS1/2* mutation, compared with *NF2*-mutant MPM and other cancer types (Figure 4A). We also provided evidence that a T-cell regulatory (Tregs) signature was enriched in *LATS1/2*-mutant MPM (Figure 4A). These data were in agreement with the above WGCNA analysis showing the presence of a mixed population of activated and exhausted T-cells related to Hippo-YAP aberration (Figure 3). Regarding B-cell infiltrates, which have been shown to play a role in promoting the anti-tumor immune response [24,47], interestingly, MPM tumors harboring *LATS1/2* rather than *NF2* display enriched plasma B-cell signature (Figure S6). Furthermore, we investigated the distribution of immune subtype models (C1–C6) across the TCGA MPM cohort [48]. Comparing the immune subtypes in low versus high protein levels of NF2 and p-YAP showed that low levels of NF2 and p-YAP are consistently associated with a lower Inflammatory (Immune C3) and higher Lymphocyte Depleted (Immune C4) signature, whereas having opposite patterns in other immune subtype signatures, such as IFN−gamma Dominant (Immune C2), TGF−beta Dominant (Immune C6), Wound Healing (Immune C1) (Figure 4B). Consistently, based on an independent MPM dataset (GSE29354) [49], we observed that the hub genes-based signature score in the red module (Figure 1I) is significantly positively correlated with YAP/TAZ target score but not NF2 gene expression (Figure 4C). Collectively, these data suggest that NF2 and p-YAP have divergent immune signatures, further supporting that NF2 has functions independent of classical Hippo-YAP signaling.

**Figure 5:**
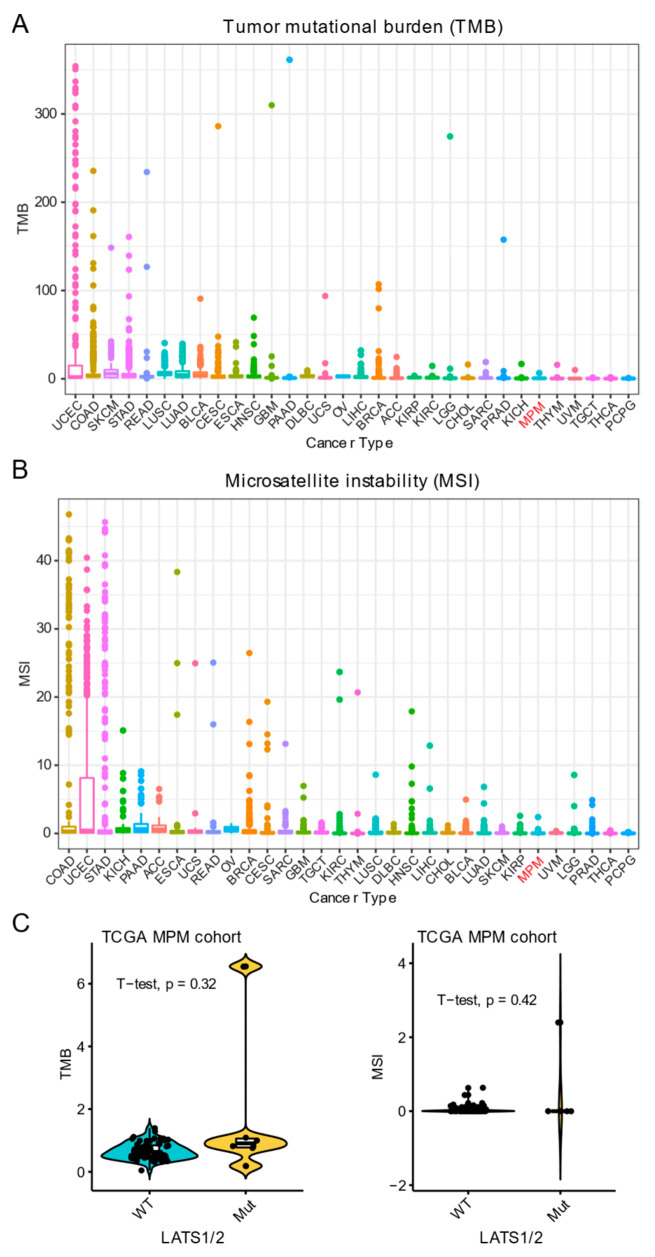
Association of *LATS1/2* mutations with TMB and MSI in MPM. (**A**,**B**) Tumor mutational burden (TMB; **A**) and microsatellite instability (MSI; **B**) across TCGA pan-cancer cohort. (**C**) Association of *LATS1/2* mutations with TMB and MSI in MPM.

PD-L1 expression, tumor mutational burden (TMB) and microsatellite instability (MSI) represent well-characterized biomarkers predicting the responsiveness to ICB treatment [50]. Notably, MPM samples display very low TMB and MSI compared with other solid cancer types (Figure 5A,B). Since the mutational status of *LATS1/2* is associated with high PD-L1 expression (Figure 2C) and predicts better responsiveness to ICB treatment (Figure 2E), we next sought to know whether *LATS1/2* mutations are related to high TMB/MSI status. Interestingly, we observed that *LATS1/2* mutations did not associate with differential TMB/MSI status compared with the WT counterparts (Figure 5C), which is in line with recent evidence demonstrating that the value of TMB in predicting the responsiveness to ICB treatment is independent of PD-L1 expression [51].

Collectively, these analyses suggested that different tumor-infiltrating immune cell patterns exist between dysregulation of NF2 and Hippo-YAP signaling in MPM.

### 2.5. NF2 and Hippo-YAP Engage Different Protein Interactors and Are Differentially Associated with Patient Prognosis

**Figure 6:**
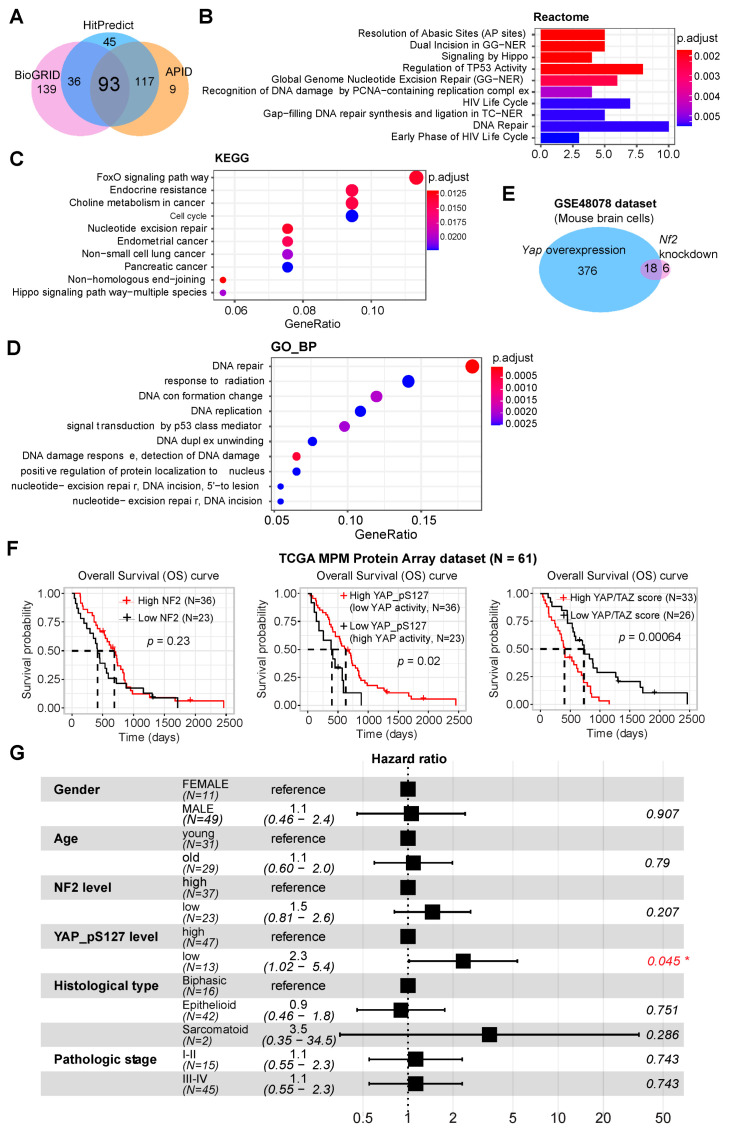
A pleiotropic role of NF2. (**A**) The common (*N* = 93) physical interactors of NF2 based on 3 curated public databases (BioGRID, HitPredict, and APID). (**B**–**D**) Top 10 significantly enriched Reactome (**B**), Kyoto Encyclopedia of Genes and Genomes (KEGG) (**C**), and Gene Ontology (GO; biological process [BP]) (**D**) pathways based on the common interactors (*N* = 93) of NF2 in (**A**). (**E**) Venn plot showing the gene changes in mouse brain cells with Yap1 overexpression and Nf2 knockout. Data were downloaded from GSE48078. (**F**) Association of NF2, phospho-YAP (S127), YAP/TAZ score with overall survival in MPM patients. (**G**) Univariate (**A**) and multivariate (**B**) survival analysis showing that YAP/TAZ score and the protein level of p-YAP (S127) but not NF2 predict prognosis of MPM patients.

Our data revealed differential roles of NF2 and YAP signaling in the pathogenesis of MPM, as indicated by different molecular signatures, drug sensitivity and immune profiles, indicating that NF2 may act through mechanisms independent of Hippo-YAP. We hypothesized that NF2 might interact with additional signaling pathways independent of the canonical Hippo-YAP. To test this, we integrated three publicly curated databases of protein physical interactions (BioGRID; HitPredict; APID) (Figure 6A), which showed that the common proteins physically interacting with NF2 are mainly enriched in DNA repair and cell cycle beside the Hippo pathway (Figure 6B–D and Figure S7). Of note, the aforementioned EGFR family members (EGFR, ERBB2) were also represented (Figure S7). Furthermore, we mined a dataset (GSE48078) [52] in which Yap1 overexpression and Nf2 knockout were performed on the same mammalian brain cells, respectively (Figure 6E), demonstrating a difference in gene expression changes (adjusted *p*-value < 0.05) resulting from Yap overexpression compared with Nf2 deletion. Clinically, univariate and multivariate survival analysis showed that YAP/TAZ score and the protein level of p-YAP but not NF2 were prognostic in MPM patients (Figure 6F,G).

Together, these data supported the notion that NF2 is engaged in additional signaling pathways beyond Hippo-YAP, which might explain the differential roles of NF2 and Hippo-YAP in the pathogenesis of MPM.

## 3. Discussion

It is a long-held notion that loss-of-function mutations in negative regulators of the Hippo pathway, such as NF2, LATS1/2, have a similar potential to promote nuclear YAP activity [11,53,54,55]. Genetic alterations of NF2 and LATS1/2 have been observed particularly in MPM, as well as tumors arising from the nervous system [4,11,12]. Evidence shows that the presence of nuclear YAP is strongly associated with mutations in NF2 in sporadic tumors that derive from the nervous system, such as schwannomas and meningiomas [12,56]. However, whether loss-of-function in these individual regulators uniformly affects the Hippo-YAP activity and contributes to a similar disease phenotype has not yet been revealed in MPM, in which Hippo pathway deregulation is frequent and thought to be critical for the pathogenesis of MPM [11]. Surprisingly and interestingly, we found in this study that loss-of-function in the core component LATS2, rather than the upstream regulator NF2 of the Hippo pathway, is linked to the aberrant activation of Hippo-YAP signaling. Integrated analysis of proteomic and transcriptomic datasets uncovered different modules/clusters associated with NF2 and p-YAP. More importantly, our findings revealed potentially targeted- or immune-therapies for MPM subsets associated with Hippo-YAP dysregulation.

### 3.1. A Pleiotropic Role of NF2

*NF2* is one of the most frequently mutated genes regulating the Hippo pathway in MPM [14]. NF2 has been shown to regulate signaling pathways other than Hippo, such as EGFR-RAS-ERK, and mediate signaling events between the actin cytoskeleton and the plasma membrane [11,13]. These observations raise the question to what extent NF2-mediated tumor suppression is mediated by the Hippo-dependent modulation of YAP/TAZ activity and the possibility that dysregulation of Hippo-independent functions of NF2 also contributes to mesothelioma development.

In this study, we showed that genetic alterations of *NF2*, despite decreasing the NF2 protein level, is not associated with altered expression of p-YAP. Furthermore, there is no correlation between NF2 and p-YAP. Pathway analyses (GO, KEGG, Reactome) revealed that the molecular clusters correlated with NF2 protein level are involved in multiple biological processes, such as MAPK, interleukin, and inflammatory signaling pathways. Additionally, the proteins physically interacting with NF2 are enriched in multiple pathways including Hippo. These results support the notion that NF2 has other functions beyond the canonical Hippo-YAP pathway in the development of MPM [11,13].

Intriguingly, we showed that solid cancer cell lines with NF2 loss-of-function may benefit from EGFR-targeted therapy (Figure 2G), which is supported by a previous study demonstrating that Nf2 deletion promotes EGFR activation, thereby rendering sensitivity of Nf2-deficient cells to EGFR inhibitors [27]. The presence of activated EGFR signaling has been reported in a subset of MPM, although the subset has not been specified [57,58,59], arguing the need for biomarker-driven stratification for EGFR-targeted therapy in MPM.

### 3.2. Hippo Pathway Dysregulation

Aberrant Hippo signaling pathway has been widely observed in clinical MPM samples [3,60]. In our study, LATS2 mutations, accounting for approximately 11% of MPM and functioning as one of the core components of the Hippo pathway, is associated with aberrant Hippo-YAP (Figure 1F). Of note, it has been reported that Hippo-YAP accounts for more than 70% of clinical MPM samples [61], which can be explained by the intersection of the Hippo signaling pathway with other pro-tumorigenic signaling pathways. The Hippo pathway is engaged in multiple biological processes, such as cell size and proliferation, immunity, metabolism, cancer therapy resistance and metastasis [7]. Emerging evidence has supported the role of canonical Hippo-YAP signaling in cancer immunity [62,63,64,65], which was also revealed in MPM in our study. Of note, the CD8+ T-cell-related immune signature is mostly dysregulated in MPM with the *LATS1/2* mutation that leads to Hippo-YAP activation, compared with other cancer types (Figure 4A). Whether this link is tumor-specific warrants further investigation.

A previous study showed that 5 out of 7 *LATS2*-mutant MPM also have co-mutated NF2, and defined a unique subset of MPM with *LATS2/NF2* co-occurring mutations [66]. Interestingly, single inactivation of either NF2 or LATS2 did not affect the proliferation of MPM cells, which, however, could be effectively enhanced by co-mutations. Moreover, based on their protein array data that contained phosphorylation levels of 40 proteins, *LATS2/NF2* co-occurring mutations displayed different molecular rewiring and therapeutic vulnerabilities compared to the single inactivation, suggesting different roles of NF2 and LATS2 in MPM. However, they did not associate this genetic background with immune infiltration.

### 3.3. Compromised Tumor Immunity in MPM

Immune checkpoint blockade (ICB) has shown promises in MPM, but is plagued with low and heterogeneous response rates [67,68,69], suggesting that biomarker-guided stratifications of MPM subsets for immunotherapies are urgently needed [1]. In a recent study, we have demonstrated that *LATS1/2* mutational status may represent a useful biomarker to stratify MPM patients for immunotherapy targeting the immune checkpoint proteins PD1/PD-L1 [9]. Previous evidence also demonstrated that LATS1/LATS2 deletion in tumors, which activates YAP signaling, induced increased tumor immunogenicity, leading to tumor destruction by enhancing anti-tumor immune responses [62]. Moreover, the YAP-driven subtype of small-cell lung cancer displays a highly enriched T-cell inflamed signature [70]. Notably, in the study by Moroishi, T. et al. [9] demonstrating the role of the *LATS1/2* mutation in suppressing immunity in cancer, their findings were based on three different murine syngeneic tumor models (B16, SCC7, and 4T1). However, in our study, we observed the role of the *LATS1/2* mutation in modulating cancer immunity is the most prominent in human MPM compared with other cancer types (Figure 4A), suggesting tissue-specific immunoregulation [71,72,73]. In this study, of particular interest, we observed that aberrant NF2 and Hippo-YAP pathways are associated with different patterns of dysregulation in adaptive immune responses. Specifically, NF2 loss-of-function is correlated with compromised BCR-mediated immunity, whereas aberrant Hippo-YAP is correlated with enriched T-cell-mediated immunity and plasma B-cell infiltrates. These lines of evidence support ICB treatment for the MPM subset with an aberrant Hippo-YAP signaling pathway. Additionally, in our study, we found exhausted adaptive immunity accompanied by YAP activation, as shown by upregulated (Figure 3C,F) or positive correlation with (Figure S4A) PD-L1 expression. In line with this, a recent study showed that the Hippo pathway could upregulate PD-L1, thereby promoting immune evasion in human cancer [74]. Together, the evidence above indicates that YAP signaling induces activated and exhausted T-cells, both prioritizing anti-PD1/PD-L1 for this MPM subset.

Currently, biomarkers to predict a clinical response have largely focused on the T-cell compartment. However, other immune subsets may also contribute to anti-tumor immunity. Activated B-cells can release antibodies that tag tumor cells for attack by other cellular players of the immune system, e.g., enabling the T-cells to target tumor cells effectively [75]. Recent studies have shown that B-cells are an essential element to predict effective antitumor immune response [24,47], highlighting the potential role of B-cells in coordinating the response, in part, to ICB treatment, with implications for the development of biomarkers and therapeutic targets. In this study, importantly, we showed that in MPM with low NF2 expression, a high plasma B-cell infiltrative signature predicts longer survival than that of a low one (Figure 2F). In addition, the mechanism whereby NF2 loss-of-function drives a deficient B-cell-mediated immunity remains unclear. Our analysis provided some potential clues through the identification of hub genes that may play a central regulatory role. Specifically, POU2AF1 and SPIB are transcription factors that are essential for the response of B-cells to antigens [18,19]. In this case, there might be a potential link among NF2, POU2AF1, and SPIB, which requires further investigation. Together, our data provide potential insights into how B-cell-mediated immune response work in the context of MPM development, which may be valuable in the design of vaccines for the MPM subset with intact NF2.

### 3.4. Combined Targeted Therapy and Immunotherapy for an MPM Subset

Recently, several studies have demonstrated the potential of immunotherapies to treat patients with mesothelioma [67,76,77]. Despite the promise, the majority of MPM patients are unresponsive to monotherapy or dual immunotherapies. PD-L1 protein expression on tumor or immune cells represents the first clinically approved predictive biomarker for the benefit of ICB treatment. In our study, we found that Hippo-YAP activity is significantly correlated with PD-L1 expression in MPM samples, supporting anti-PD/PD-L1 immunotherapy for this subset of MPM patients. More intriguingly, we found that mutations affecting the core signaling molecules (LATS1/2, MST1) regulating Hippo-YAP activity in MPM patients show better overall survival after ICB treatment.

There is a growing interest in the development of combined immunotherapy and molecularly targeted therapies in fighting against cancer [78,79,80]. In the setting of MPM, how to optimize combination therapy remains a challenge. In this study, we found that inhibitors selectively targeting BCR-ABL, such as Dasatinib, preferentially inhibit *LATS1/2*-mutant MPM cells. More importantly, Dasatinib and other clinically approved BCR-ABL inhibitors have been shown to enhance T-cell-mediated immune response in various cancer types [9,39,81], including MPM [81]. Based on multi-region whole-exome and T-cell receptor (TCR) sequencing, Chen et al. [81] evaluated the evolution of T-cell repertoire heterogeneity of MPM under Dasatinib treatment, demonstrating a highly heterogeneous TCR repertoire within MPM samples. Intriguingly, the evidence also showed that, compared to pre-treatment tumors, Dasatinib treatment induced a significant increase in T-cell clonality, and patients with higher T-cell clonality and more homogeneous T-cell repertoire after treatment had significantly better survival. The findings by Chen and colleagues indicated that Dasatinib might induce expansion and reactivation of T-cells, highlighting combined ICB with Dasatinib as a promising new treatment for MPM. However, the study by Chen et al. did not investigate their findings with Dasatinib in the context of genetic alterations in MPM.

Collectively, the combination of BCR-ABL targeted therapies with ICB might enhance the clinical utility of immunotherapy for MPM subsets with YAP activation. Additionally, considering the association between NF2 loss-of-function and deficient BCR signaling and the crucial role of B-cells in enhancing T-cell-mediated anti-cancer immunity [24,47], the combination of intact NF2 with activated YAP signaling pathways may provide a better stratification of MPM patients responsive to ICB.

### 3.5. Study Limitations

This study contains some limitations, e.g., the lack of experimental and cohort validations of the findings, and small sample size.

## 4. Materials and Methods

### 4.1. WGCNA and Function Enrichment Analyses

To identify the gene expression profiling associated with the protein level of NF2 and p-YAP (S127) in MPM, we applied the R package “WGCNA” to whole-genome transcriptomic data of the TCGA MPM cohort. In WGCNA, genes are clustered based on the co-expression patterns, then a gene co-expression network is constructed, which was transformed into the adjacency matrix and then topological overlap matrix (TOM) [9,10]. Genes were grouped into different modules (clusters) using the dynamic tree cut algorithm, according to the TOM-based dissimilarity. The module eigengene (ME) was calculated based on the first principal component of each module. The ME values were correlated (Pearson) with sample traits defined by the protein level of NF2 and p-YAP (S127) in MPM samples. Here, we set the soft-thresholding power at 12 (scale-free R^2^ = 0.9), cut height at 0.25, and minimal module size to 30, to identify key modules. The module significantly correlated with sample traits was selected to explore its biological functions, such as Gene Ontology (GO), Kyoto Encyclopedia of Genes and Genomes (KEGG) and Reactome pathway enrichment analyses, using the R package “clusterprofiler” [82]. Hub genes were defined as the top 30 intramodular connected genes.

### 4.2. Cell Culture and Cell Viability Assay

Cell lines used in this study include: normal human mesothelial cells Met-5A (RRID: CVCL_3749), MPM cell lines NCI-H28 (from ATCC (American Type Culture Collection, Manassas, VA, USA); RRID: CVCL_1555), NCI-H2052 (from ATCC; RRID: CVCL_1518), NCI-H2452 (from ATCC; RRID: CVCL_1553) [9,83], ACC-MESO-4 (from RIKEN Cell Bank; RRID: CVCL_5114), ACC-MESO-1 (from RIKEN Cell Bank (Ibaraki, Japan); RRID: CVCL_5113), MSTO-211H (DSMZ (German Collection of Microorganisms and Cell Cultures, Brunswick, Germany); RRID: CVCL_1430) and JL-1 (DSMZ; RRID: CVCL_2080), and primary MPM cells (BE261T, established from surgically resected MPM tumors). All human cell lines have been authenticated using STR profiling within the last three years, and are confirmed free from mycoplasma contamination (Microsynth, Bern, Switzerland). Cells were cultured in RPMI-1640 medium with 10% fetal bovine serum and 1% penicillin/streptomycin. The human study was performed under the auspices of protocols approved by the institutional review board of Inselspital Bern (KEK number: 042/15), and informed consent was obtained from patients.

Cells seeded in triplicate at 96-well plates (1000–1500 cells/well in the tissue-culture treated plate (Corning, #353072)) were treated with the indicated drugs the next day, over a 12-point concentration range (two-fold dilution), with DMSO as the vehicle. Cell viability was determined 96 h post-treatment by the Acid Phosphatase Assay Kit (ab83367; Abcam) [9,84]. The median inhibitory concentration (IC50) was calculated using GraphPad Prism 8.

### 4.3. Immunoblots

Cell lysates were prepared using RIPA buffer (Cell Signaling Technology) containing protease inhibitor cocktail and phosphatase inhibitors. Equal amounts of total proteins were separated by SDS-PAGE (#4561033; Bio-Rad) and transferred to nitrocellulose membranes (#170-4156; Bio-Rad). After brief incubation with blocking buffer (#927-4000; Li-COR Biosciences, Bad Homburg, Germany) at room temperature, the membranes were blotted with primary antibodies (LATS1 (Cell Signaling Technology, #9153S), LATS2 (Novus Biologicals, #NBP3-03913), NF2, (Cell Signaling Technology, #6995S), beta-actin (Cell Signaling Technology, #3700S)) and anti-rabbit (#926–32211) or anti-mouse (#926-68020) secondary antibodies (Promega, Madison, WI, USA). Membrane-bound secondary antibodies were visualized by the Odyssey Infrared Imaging System (Li-COR Biosciences).

### 4.4. Public Databases

Transcriptomic (*n* = 87) and whole-genome exome sequencing data (*n* = 81) of MPM samples were downloaded from TCGA (https://portal.gdc.cancer.gov/ accessed on 21 December 2020). The clinical characteristics of MPM patients were summarized in Table S1. Normalized level 3 data of reverse phase protein array (RPPA) were downloaded from The Cancer Proteome Atlas (TCPA) database (https://tcpaportal.org/tcpa/ accessed on 21 December 2020) [85], which quantified 220 proteins in 61 out of the 87 MPM samples in TCGA. Here, TCPA Level 3 data were utilized, and the proteomic data normalization was processed as follows (For the details please refer to the FAQ section: https://tcpaportal.org/mclp/# accessed on 21 December 2020): (1) Calculate the median for each protein across all the samples. (2) Subtract the median (from step 1) from values within each protein. (3) Calculate the median for each sample across all proteins. (4) Subtract the median (from step 3) from values within each sample.

Protein-interacting data were downloaded from Agile Protein Interactomes DataServer (http://cicblade.dep.usal.es:8080/APID/init.action accessed on 21 December 2020), BioGRID (version 4.0; https://thebiogrid.org/ accessed on 21 December 2020), and HitPredict (http://www.hitpredict.org/ accessed on 21 December 2020). The interaction map among the interactors of NF2 was constructed and downloaded from STRING (https://string-db.org/ accessed on 21 December 2020) [86]. Drug (*n* = 481) sensitivity data across solid cancer cell lines (*n* = 659) were downloaded and reanalyzed from published studies [87,88]. Fisher’s z-transformation was applied to the correlation coefficients to adjust for (normalize) variations in cancer cell line numbers across small molecules and cell lineages. Genetic and survival data of patients after immunotherapies (anti-PD1/PDL1, anti-CTLA4) were from a TMB and immunotherapy (MSKCC) cohort in cBioPortal (https://www.cbioportal.org/ accessed on 21 December 2020). Tumor-infiltrating immune cell profiles across TCGA pan-cancer cohort were downloaded from TIMER (version 2.0), a comprehensive resource for systematic analysis of immune infiltrates across diverse cancer types (http://timer.comp-genomics.org/ accessed on 21 December 2020) [46]. Immune subtype models (C1–C6) were based on a previous study [48]. The genes contained in each signature were evaluated using model-based clustering by p the “mclust” R package. Each sample was finally to be grouped based on its predominance with the C1-C6 signature. Data of mouse brain cells with Yap1 overexpression and Nf2 knockout were downloaded from GSE48078 [52]. R packages “limma” and “edgeR” were used to normalize the data and identify the differential gene or protein expression, respectively.

### 4.5. Gene Signature Scores

#### 4.5.1. YAP-TAZ Target Gene Signature Score

A curated 22-gene YAP-TAZ target gene signature (MYOF, AMOTL2, LATS2, CTGF, CYR61, ANKRD1, ASAP1, AXL, F3, IGFBP3, CRIM1, FJX1, FOXF2, GADD45A, CCDC80, NT5E, DOCK5, PTPN14, ARHGEF17, NUAK2, TGFB2, RBMS3) was utilized based on a previous study [15], which used published RNA-sequencing and ChIP-sequencing data across various cancer types. YAP-TAZ target score was calculated by summarizing the Z-normalized log2RSEM (RNA-Seq by Expectation-Maximization) of the expression data for the 22 curated YAP/TAZ downstream transcription target genes, based on the TCGA MPM RNA-sequencing data.

#### 4.5.2. Hub Genes-Based Signature Score

Hub genes-based signature score was generated using the top 30 connected genes (LCK, CD3E, SH2D1A, CD6, CD3D, SIRPG, CD2, SLAMF6, IL2RG, CD247, TBC1D10C, TRAT1, SIT1, ZAP70, CXCR3, SLA2, ITGAL, CD5, GZMK, TIGIT, CD96, ZNF831, ITK, THEMIS, PYHIN1, P2RY10, ACAP1, CXCR6, CD27, GPR171) in the red module. The signature score was calculated by summarizing the Z-normalized log2RSEM of the gene expression data and then applied to an independent MPM dataset (GSE29354) from NCBI Gene Expression Omnibus (GEO) data repository portal [49]. R packages “limma” and “edgeR” were used to normalize the data and identify the differential gene or protein expression, respectively [84].

### 4.6. Survival Analysis

Survival analysis was performed using “survminer” and “survival” R packages. Tumor samples within the TCGA MPM cohort were divided into two groups, based on each hub gene’s best-separation cut-off value to plot the Kaplan–Meier survival curves.

### 4.7. Statistics

Data were presented as mean ± SD, with the indicated sample size (n) representing biological replicates. Gene expression and survival data derived from the public database, as well as the correlation coefficient, were analyzed using R (version 3.6.3). *p* < 0.05 was considered statistically significant.

## 5. Conclusions

Overall, our results suggest that NF2 loss-of-function and dysregulated Hippo-YAP signaling have different molecular aberrations and therapeutic implications in MPM. Based on these findings, we propose that MPM patients with aberrant Hippo-YAP pathway, e.g., determined by IHC staining of clinical MPM samples or genetic alteration of *LATS1/2*, may benefit from combined treatment of BCR-ABL-targeted therapies and ICBs, such as combined Dasatinib and anti-PD-L1 treatment.

## Data Availability

The data presented in this study are available in this article (and Supplementary Material).

## Supplementary Materials

The following are available online at https://www.mdpi.com/article/10.3390/cancers13071561/s1, Figure S1: DNA methylation profiles of major genes regulating the Hippo signaling pathway across TCGA MPM samples. The *X*-axis represents the methylation probes (450k array) recognizing different CpG sites of the indicated genes, Figure S2: Functional analysis of genes enriched in greenyellow and blue modules. A, B, Top 5 significantly enriched Gene Ontology (GO; biological process [BP]), Kyoto Encyclopedia of Genes and Genomes (KEGG) (B), and Reactome (C) pathways based on genes in the greenyellow (A) and blue (B) modules. Cnetplot in (A, lower panel) listed genes in the enriched Reactome pathways, Figure S3: Functional analysis of genes enriched in turquoise and green modules. A, B, Top 5 significantly enriched Gene Ontology (GO; biological process (BP)), Kyoto Encyclopedia of Genes and Genomes (KEGG) (B), and Reactome (C) pathways based on genes in the turquoise (A) and green (B) modules. Cnetplot in (A, lower panel) listed genes in the enriched Reactome pathways, Figure S4: MPM tumors display the highest correlation pattern between YAP/TAZ downstream target score and PD-L1 expression. A, Correlation (Spearman) analysis between the YAP/TAZ downstream target score and PD-L1 expression across the TCGA Pan-cancer cohort. Of note, MPM tumors display the highest correlation pattern. * indicates a significantly (*p* < 0.05) positive correlation; # represents a significantly negative correlation. B, Barplots showing the YAP/TAZ downstream target score, which reflects the activity of the YAP signaling pathway, across the TCGA Pan-cancer cohort. Of note, MPM tumors display the highest YAP/TAZ downstream target score, Figure S5: Immunoblots of MPM cells showing the expression of LATS1, LATS2, and NF2; Actin as an internal control, Figure S6: Tumor-infiltrating B-cell profiles across the TCGA pan-cancer cohort based on NF2 and LATS1/2 mutational status. Data were downloaded from TIMER (version 2.0) (See the methods), Figure S7: The interaction map among the common interactors of NF2. Data were constructed and downloaded from STRING (https://string-db.org/ accessed on 21 December 2020), Table S1: Clinical characteristics of included TCGA MPM patients.
