# Supplementary material for: NF2 and Canonical Hippo-YAP Pathway Define Distinct Tumor Subsets Characterized by Different Immune Deficiency and Treatment Implications in Human Pleural Mesothelioma
Source: Cancers (Basel). 2021 Mar 29;13(7):1561. doi: 10.3390/cancers13071561 (PMC8036327; doi:10.3390/cancers13071561)
Supplement: Supplementary file 1 [file cancers-13-01561-s001.pdf]

cancers

MDPI

Supplementary materials: NF2 and Canonical Hippo-YAP Pathway Define Distinct Tumor Subsets Characterized by Different Immune Deficiency and Treatment Implications in Human Pleural Mesothelioma

Haitang Yang, Sean R. R. Hall, Beibei Sun, Liang Zhao, Yanyun Gao, Ralph A. Schmid, Swee T. Tan, Ren-Wang Peng and Feng Yao

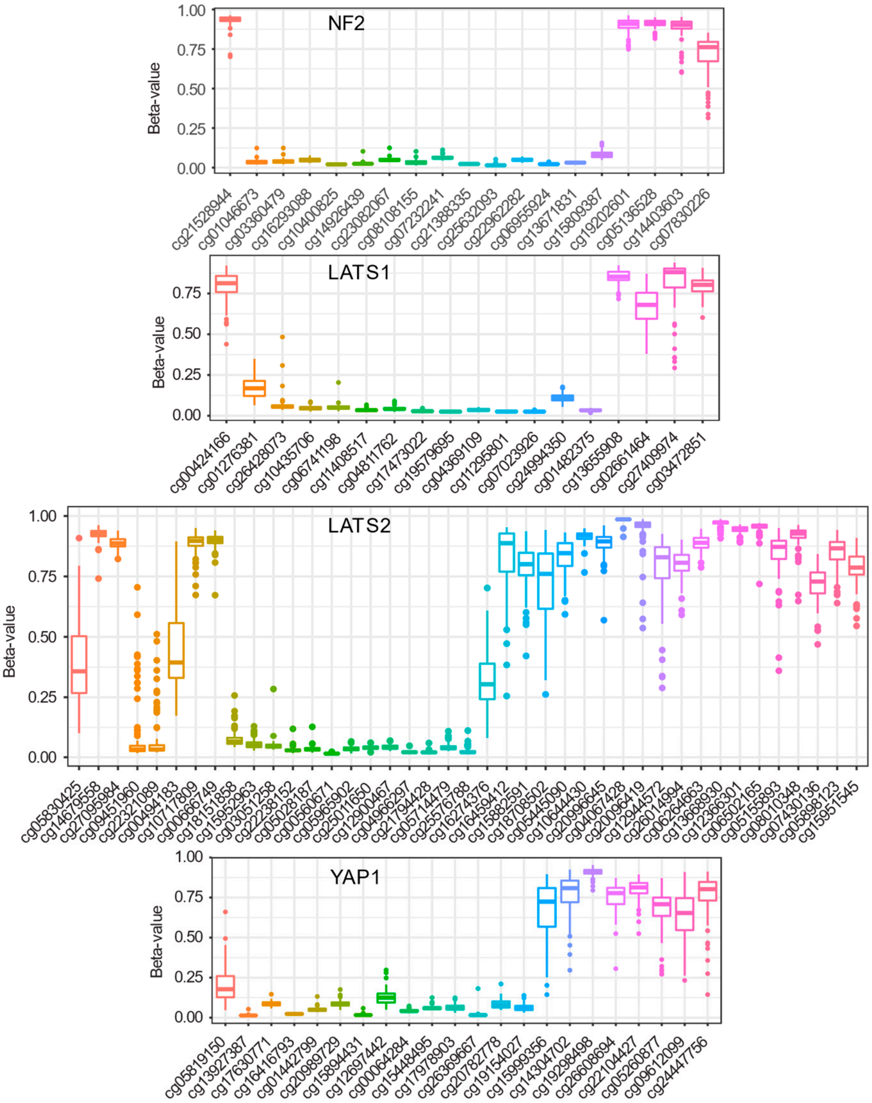

Figure S1. DNA methylation profiles of major genes regulating the Hippo signaling pathway across TCGA MPM samples. The *X*-axis represents the methylation probes (450k array) recognizing different CpG sites of the indicated genes.

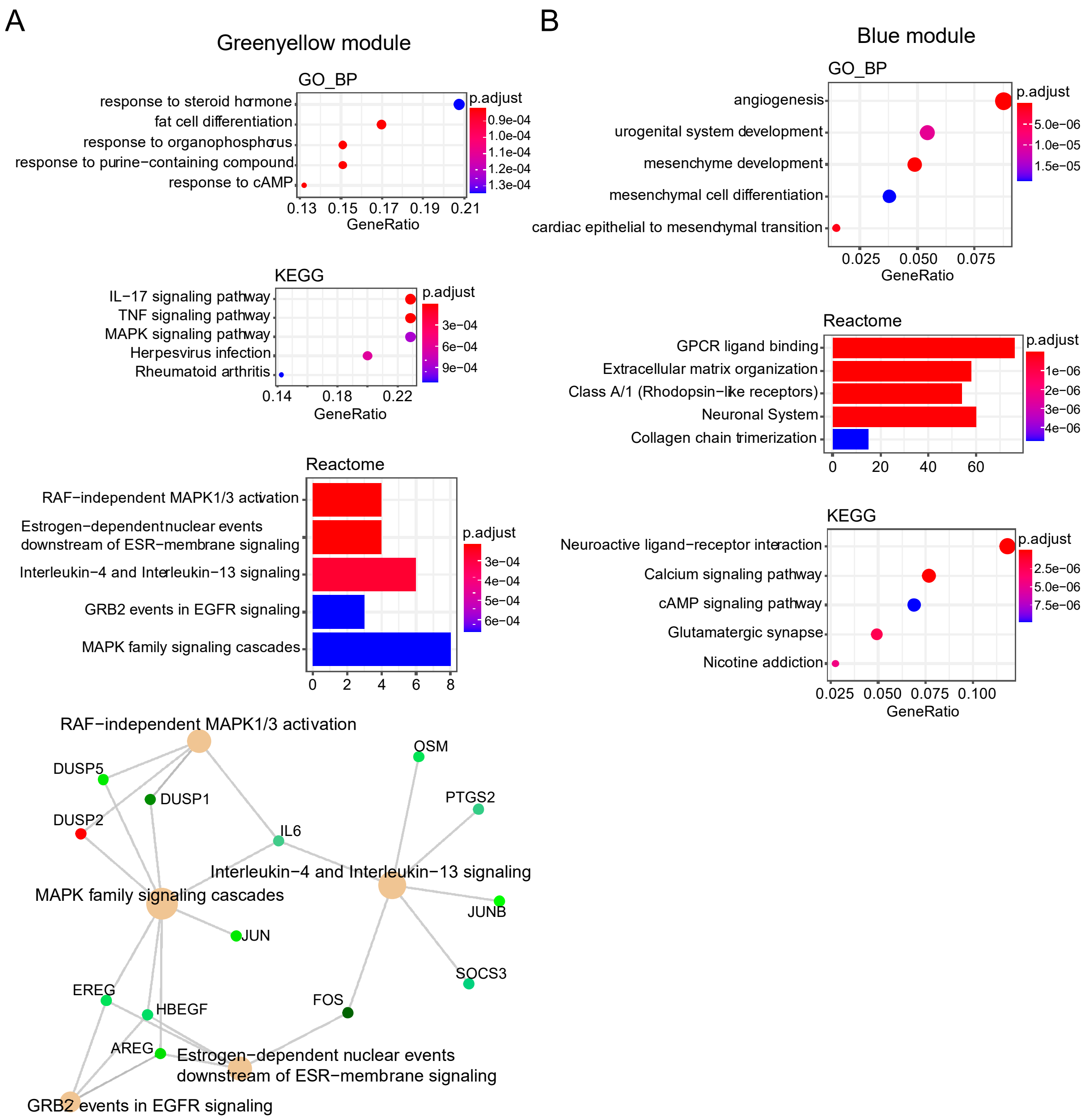

Figure S2. Functional analysis of genes enriched in greenyellow and blue modules. A, B, Top 5 significantly enriched Gene Ontology (GO; biological process [BP]), Kyoto Encyclopedia of Genes and Genomes (KEGG) (B), and Reactome (C) pathways based on genes in the greenyellow (A) and blue (B) modules. Cnetplot in (A, lower panel) listed genes in the enriched Reactome pathways.

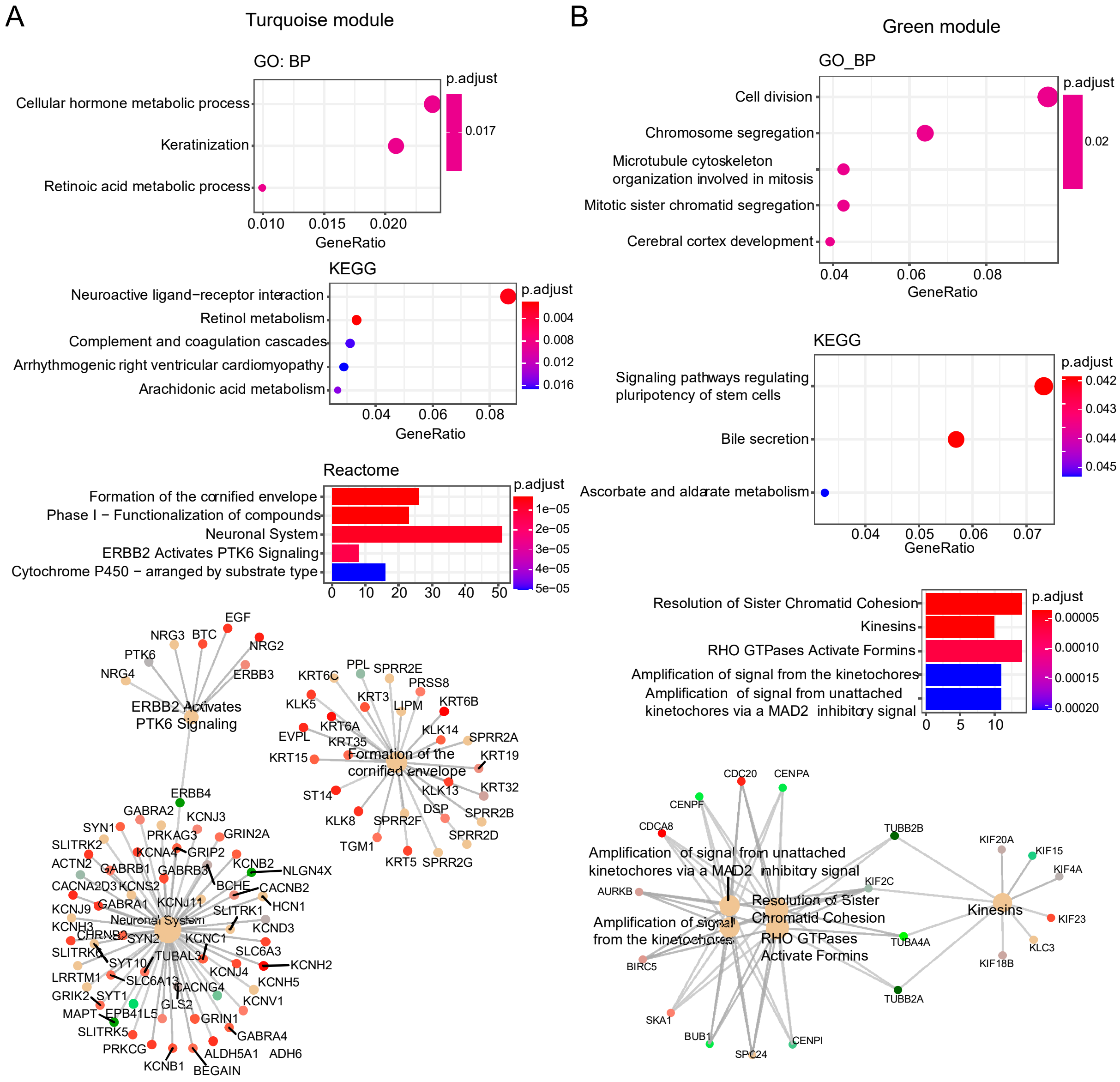

Figure S3. Functional analysis of genes enriched in turquoise and green modules. A, B, Top 5 significantly enriched Gene Ontology (GO; biological process (BP)), Kyoto Encyclopedia of Genes and Genomes (KEGG) (B), and Reactome (C) pathways based on genes in the turquoise (A) and green (B) modules. Cnetplot in (A, lower panel) listed genes in the enriched Reactome pathways.

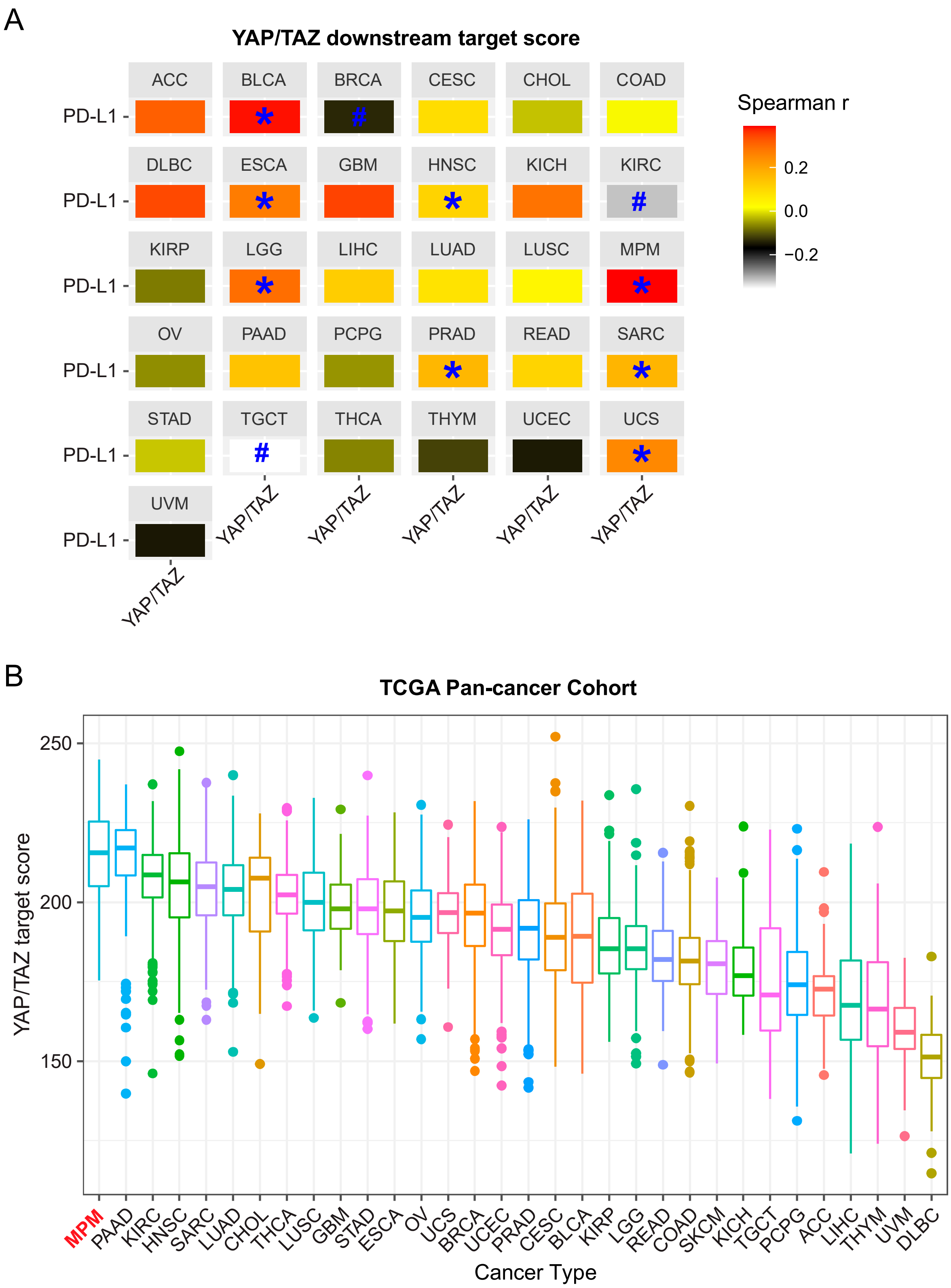

Figure S4. MPM tumors display the highest correlation pattern between YAP/TAZ downstream target score and PD-L1 expression. A, Correlation (Spearman) analysis between the YAP/TAZ downstream target score and PD-L1 expression across the TCGA Pan-cancer cohort. Of note, MPM tumors display the highest correlation pattern. * indicates a significantly ($p < 0.05$) positive correlation; # represents a significantly negative correlation. B, Barplots showing the YAP/TAZ downstream target score, which reflects the activity of the YAP signaling pathway, across the TCGA Pan-cancer cohort. Of note, MPM tumors display the highest YAP/TAZ downstream target score.

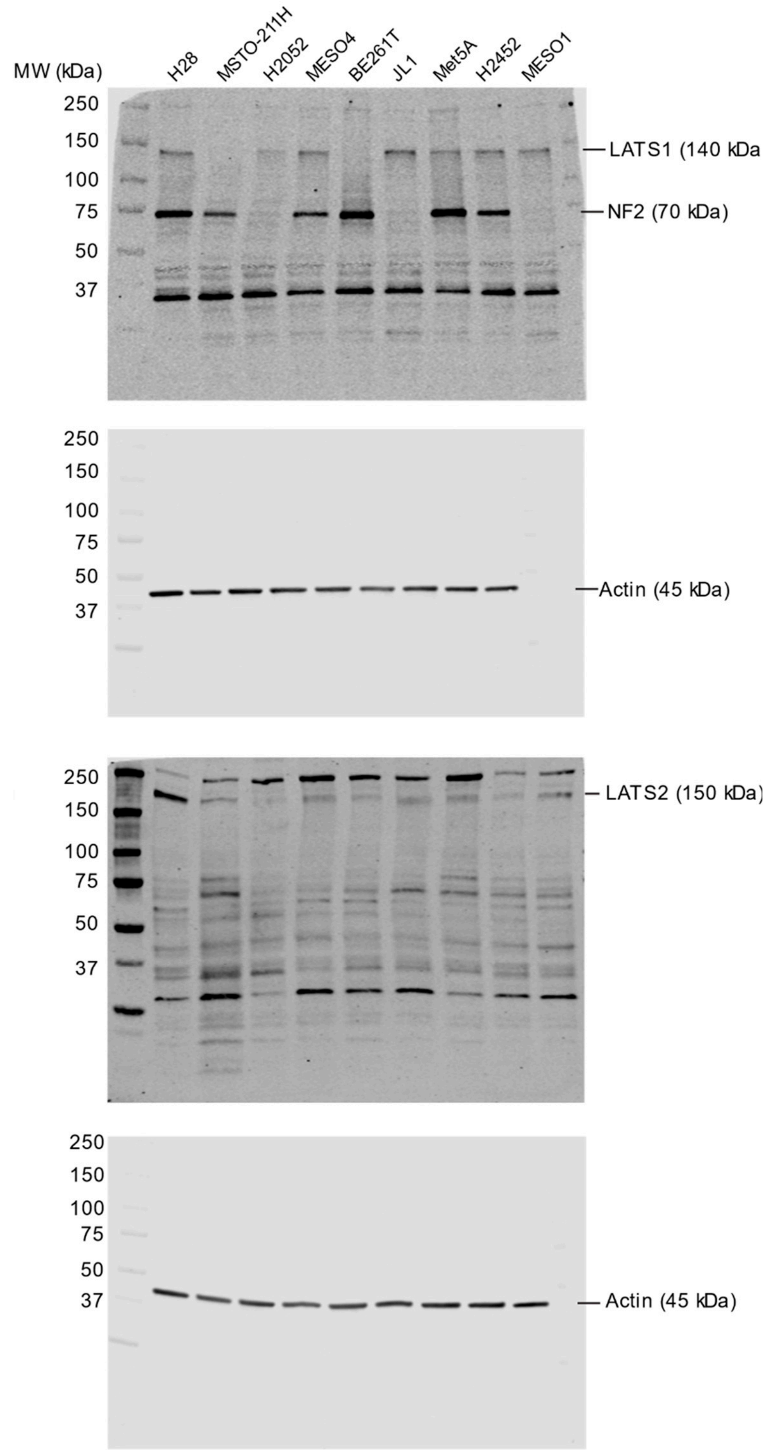

Figure S5. Immunoblots of MPM cells showing the expression of LATS1, LATS2, and NF2; Actin as an internal control.

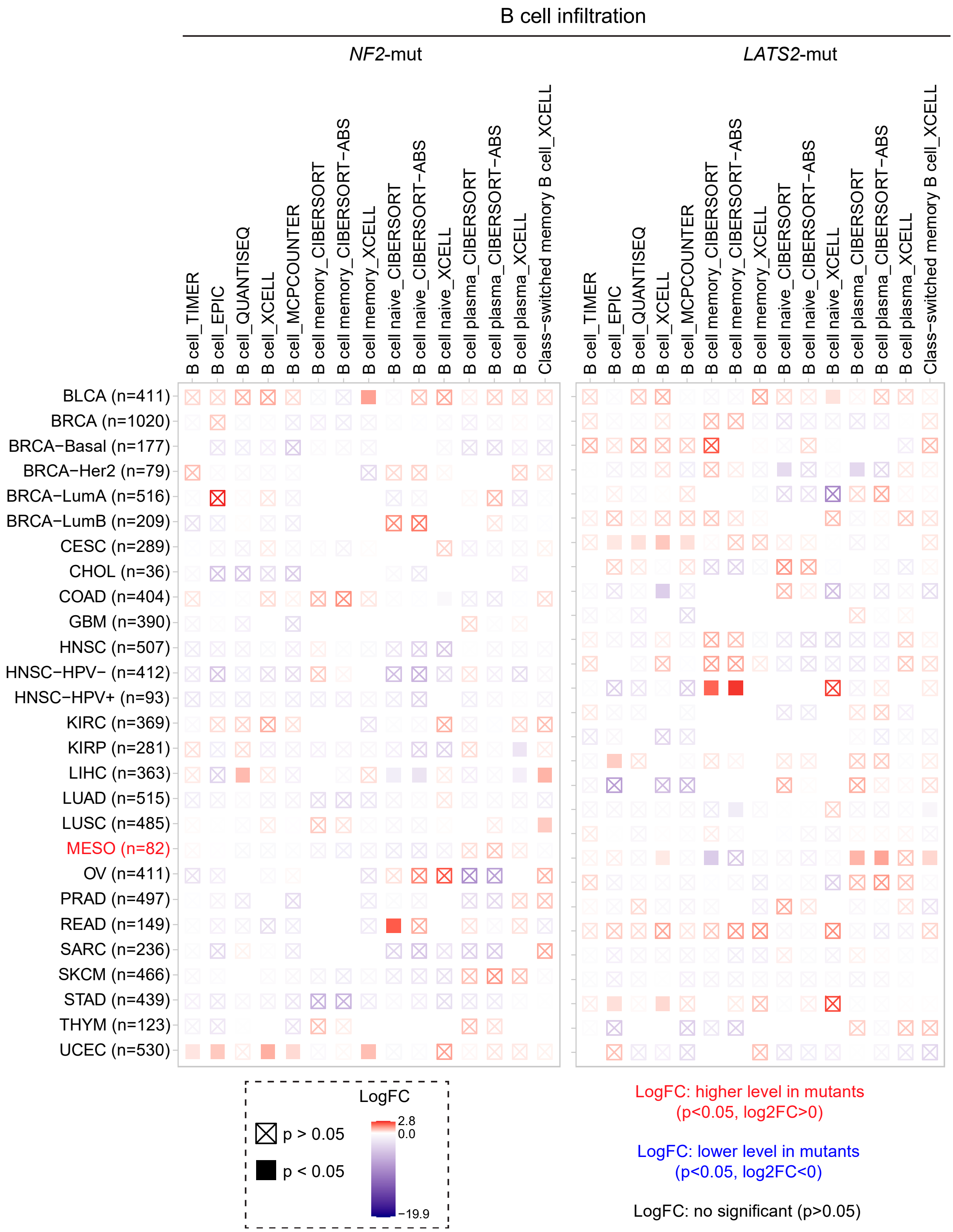

Figure S6. Tumor-infiltrating B-cell profiles across the TCGA pan-cancer cohort based on NF2 and LATS1/2 mutational status. Data were downloaded from TIMER (version 2.0) (See the methods).

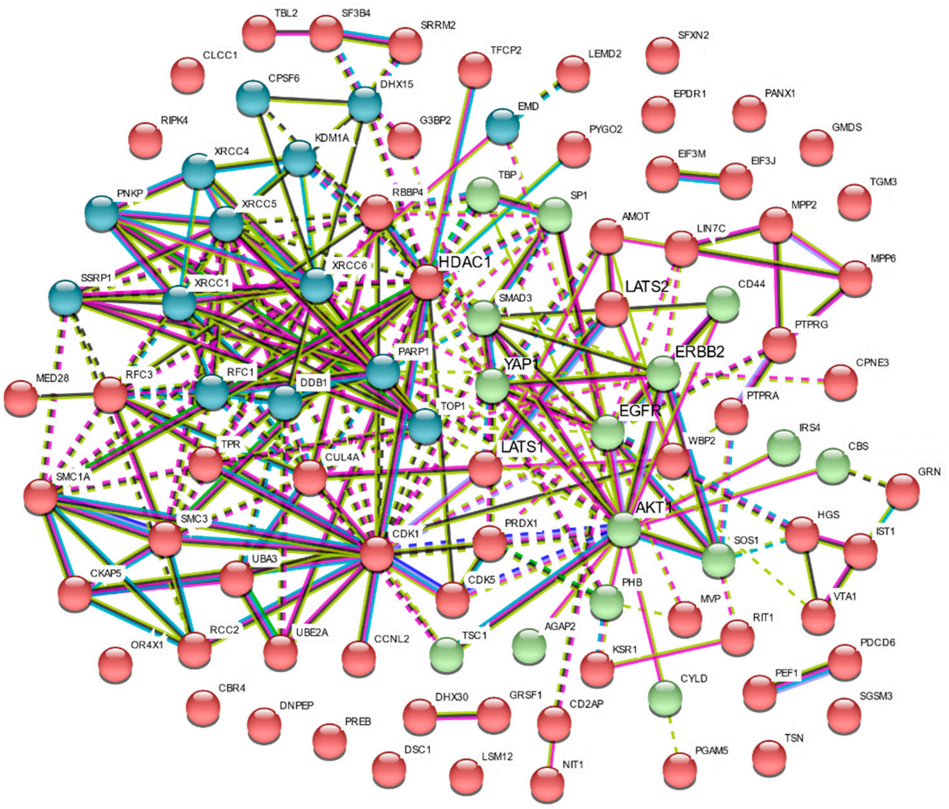

Figure S7. The interaction map among the common interactors of NF2. Data were con-structed and downloaded from STRING (https://string-db.org/).

Table S1. Patient Charactersitcs.

Clinical Characteristics	level	Overall
No.		87
Sex (%)	Female	16 (18.4)
	Male	71 (81.6)
Age (mean (SD))		62.99 (9.76)
Pathological T Stage (%)	T1	14 (16.1)
	T2	26 (29.9)
	T3	32 (36.8)
	T4	13 (14.9)
	TX	2 (2.3)
Pathological N stage (%)	N0	44 (50.6)
	N1	10 (11.5)
	N2	26 (29.9)
	N3	3 (3.4)
	NX	4 (4.6)
Pathological M stage (%)	M0	57 (65.5)
	M1	3 (3.4)
	MX	27 (31.0)
AJCC TNM (6th) Stage (%)	I	10 (11.5)
	II	16 (18.4)
	III	45 (51.7)
	IV	16 (18.4)
Histology_subtype (%)	Biphasic	23 (26.4)
	Diffuse	5 (5.7)
	Epithelioid	57 (65.5)
	Sarcomatoid	2 (2.3)
